# Meta-analysis of comparative effects of mobile app–based digital rehabilitation versus traditional rehabilitation after total knee arthroplasty

**DOI:** 10.3389/fphys.2025.1737373

**Published:** 2026-01-12

**Authors:** Liang Ma, Jia-Qiang Liu, Kun Yu, Mei-Yun Tan

**Affiliations:** 1 Department of Orthopedics, Affiliated Hospital of Southwest Medical University, Luzhou, Sichuan, China; 2 Department of Orthopedics, The Third Affiliated Hospital of Chengdu Medical College, Chengdu Pidu District People’s Hospital, Chengdu, Sichuan, China

**Keywords:** total knee arthroplasty, digital rehabilitation, traditional rehabilitation, meta-analysis, mobile apps

## Abstract

**Objective:**

The purpose of this study is to compare the effect of digital rehabilitation based on mobile app and traditional rehabilitation program after total knee arthroplasty.

**Methods:**

The following electronic databases were systematically searched to identify eligible trials: PubMed, EMBASE, Web of Science, the Cochrane Library, Scopus, and CINAHL. The searches were conducted from database inception to 1 December 2025. According to the inclusion and exclusion criteria, literature screening, data extraction and evaluation of its methodological quality were carried out. RevMan5.4 software was used to conduct heterogeneity test and meta-analysis of the included studies, and Cochrane systematic review tool was used to evaluate the literature publication bias. The main outcomes were visual analogue scale (VAS) of pain, “standing walking” timing test, knee function score (KSS), knee range of motion (ROM), and 10-m Walk Test.

**Results:**

This meta-analysis included 8 studies involving a total of 694 patients. Compared with traditional rehabilitation, the app-based group showed significantly lower pain scores, as assessed by the Visual Analogue Scale (VAS), with a positive mean difference favoring the app-based group (MD = 1.03, 95% CI 0.30 to 1.75; I^2^ = 0%; P = 0.006). Performance-based functional outcomes favored the app-based group, with significant improvements observed in the Timed Up and Go test (MD = −1.75, 95% CI −2.55 to −0.94; I^2^ = 19%; P < 0.0001) and the 10-m Walk Test (SMD = 0.47, 95% CI 0.05 to 0.88; I^2^ = 0%; P = 0.03). Knee range of motion (ROM) was also significantly greater in the app-based rehabilitation group (MD = 6.46°, 95% CI 2.92 to 10.00; I^2^ = 53%; P = 0.0004). No significant difference was observed in the Knee Society Score (KSS) between groups (P > 0.05). The overall risk of bias among the included studies was moderate, primarily due to unclear allocation concealment and lack of blinding.

**Conclusion:**

Compared with traditional rehabilitation, mobile app–based digital rehabilitation after total knee arthroplasty was associated with improved pain relief and better performance-based functional outcomes, as well as greater early gains in knee range of motion. No significant difference was observed in composite knee function scores. Given the low to moderate certainty of evidence, app-based rehabilitation may be considered as an adjunct or alternative rehabilitation strategy for selected patients, while further high-quality randomized controlled trials are needed to confirm long-term effectiveness.

## 1 Introduction

Total knee arthroplasty (TKA) is a widely performed surgical intervention globally and represents an effective treatment for advanced knee osteoarthritis (OA). Over the past 2 decades, the annual incidence of TKA has increased steadily on a global scale (Yelin et al., 2016), with reported rates of approximately 150–200 procedures per 100,000 inhabitants in developed countries (den Hertog et al., 2012). As the number of TKA procedures continues to rise, optimizing postoperative rehabilitation strategies has become essential to improve functional recovery and patient satisfaction.

In addition to conventional on-site physical therapy, several alternative rehabilitation strategies have been proposed after knee arthroplasty, including telerehabilitation, neuromuscular electrical stimulation, guided home-based therapy, and application-based muscle training (Klika et al., 2022; Peng et al., 2021; Zhao et al., 2023; Shukla et al., 2017). With advances in digital health technology, mobile application–based rehabilitation has emerged as a specific and increasingly popular form of digital rehabilitation after TKA. Unlike general telehealth systems or hardware-dependent platforms, mobile applications enable patients to access rehabilitation guidance, exercise programs, and symptom monitoring directly through smartphones (Shukla et al., 2017; Moffet et al., 2015; Fillingham et al., 2018; Fleischman et al., 2019; Wang et al., 2019; Hardt et al., 2018; Jiang et al., 2018; Ko et al., 2013; Kramer et al., 2003; Li et al., 2017; Mahomed et al., 2008; Russell et al., 2011; Tousignant et al., 2011). Randomized controlled trials have reported that app-based or smartphone-assisted rehabilitation programs can improve early postoperative outcomes, including functional scores and range of motion, compared with conventional rehabilitation following total knee arthroplasty (Bä et al., 2021; Zhao et al., 2024). Systematic evidence also suggests that mobile application–based telerehabilitation may lead to better overall functional and pain outcomes (Özden and Sarı, 2023). Furthermore, app-based platforms offer practical advantages over other forms of digital rehabilitation, including greater accessibility, flexibility, and scalability, as they require minimal equipment and can be seamlessly incorporated into patients’ daily lives (Krištof Mirt et al., 2025; Nussbaum et al., 2018; Ryan et al., 2022; Quach, 2024).

Several systematic reviews and meta-analyses have evaluated the effectiveness of digital or telerehabilitation interventions after total knee arthroplasty. For instance, Pastora-Bernal et al. reported favorable evidence for telerehabilitation compared with conventional rehabilitation across arthroplasty patients, including knee replacements (Pastora-Bernal et al., 2017). Other reviews have synthesized outcomes of diverse telehealth formats, such as videoconferencing and remote monitoring systems, but without isolating the effects of specific mobile application–based programs (Wang et al., 2023; Tsang et al., 2024). Recently, Özden et al. conducted a systematic review and meta-analysis focused specifically on mobile application–based rehabilitation practices in TKA, yet the evidence base remains limited and heterogeneous in terms of intervention design and outcomes reported (Özden and Sarı, 2023).

Although various forms of digital and remote rehabilitation have been studied, there remains ongoing debate regarding the comparative effectiveness of mobile application–based rehabilitation versus traditional rehabilitation following TKA. Moreover, despite the growing number of randomized controlled trials in this field, comprehensive meta-analyses focusing specifically on app-based digital rehabilitation compared with conventional rehabilitation remain limited. Therefore, the primary objective of the present study was to systematically collect and quantitatively synthesize available evidence comparing app-based digital remote rehabilitation with traditional rehabilitation after TKA. The findings of this meta-analysis aim to provide evidence-based guidance for postoperative rehabilitation strategies in the era of digital medicine.

## 2 Data and methodology

### 2.1 Agreement and registration

This systematic review and meta-analysis were conducted in accordance with the Preferred Reporting Items for Systematic Reviews and Meta-Analyses (PRISMA) guidelines and was prospectively registered in the PROSPERO database (CRD42024516546; registered on 4 March 2024).

### 2.2 Inclusion and exclusion criteria

#### 2.2.1 Inclusion criteria

① Study design: Only randomized controlled trials (RCTs) were eligible for inclusion. Quasi-experimental studies, non-randomized trials, and observational studies were not considered eligible for this review. ② Population: patients who underwent total knee arthroplasty (TKA), regardless of age, sex, race, nationality, or disease duration. ③ Intervention and comparison: studies comparing mobile application–based rehabilitation interventions with conventional or traditional rehabilitation programs. ④ Outcomes: at least one clinically relevant outcome in the domains of pain, knee function/physical performance, or range of motion (ROM) (e.g., VAS/NRS for pain; KSS/OKS/WOMAC or performance-based tests such as the Timed Up and Go test or 10-m walk test for function; knee ROM measured in degrees). These outcomes were selected based on their clinical relevance and their frequent use in previous rehabilitation studies following TKA.

#### 2.2.2 Exclusion criteria

① Duplicate publications, case reports, letters, reviews, conference abstracts, non-human and physical experimental studies, systematic reviews and meta-analyses were excluded; ② Studies with unclear eligibility criteria, inappropriate study design for the review question, or insufficient information to judge eligibility were excluded. ③ Studies were excluded if the full text could not be obtained or if outcome data were insufficient for qualitative or quantitative synthesis.

### 2.3 Document retrieval strategy

A comprehensive literature search was conducted in PubMed, EMBASE, Web of Science, the Cochrane Library, Scopus, and CINAHL from database inception to 1 December 2025. We conducted a thorough search of pertinent databases, including six English electronic databases. Chinese electronic databases were not searched because preliminary screening indicated a lack of relevant randomized controlled trials in this field. Both subject headings and free-text terms were used for database searches. When required data were missing or unclear, the corresponding authors were contacted by email; however, no responses were received. As an illustration, the English search phrases employed in PubMed encompass “mobile applications,” “rehabilitation,” and “arthroplasty, replacement, knee”. Table 1 lists the detailed search strategies and search words of the PubMed database. The full search strategies for all databases are available in the Supplementary Material (Supplementary Table S1).

**TABLE 1:** Search strategy in PubMed.

Search	Query	Results
#1	(“Arthroplasty, replacement, Knee” [Mesh] OR “total knee arthroplasty” OR “total knee replacement” OR TKA OR TKR OR “knee arthroplasty”)	53564
#2	“Rehabilitation” [Mesh] OR rehabilitation OR physiotherapy OR “physical therapy” OR exercise OR “exercise therapy” OR telerehabilitation OR “remote rehabilitation” OR “home-based rehabilitation”	1475593
#3	(“Mobile Applications” [Mesh] OR “mobile application*” OR app OR apps OR smartphone* OR health OR “digital rehabilitation” OR “app-based”)	7784520
#4	(“Randomized controlled trials as Topic” [Mesh] OR “randomized trial” OR “randomized controlled trial” OR RCT)	917627
#5	#1 AND #2 AND #3	599

### 2.4 Document screening and data extraction

All retrieved records were imported into EndNote for duplicate removal. After deduplication, title and abstract screening as well as full-text eligibility assessment were independently performed by two reviewers according to the predefined inclusion and exclusion criteria. Disagreements were resolved through discussion with a third reviewer. Data extracted included author, publication year, study design, sample size, demographic characteristics, intervention details, and reported outcomes.

### 2.5 Risk of bias assessment

Risk of bias was independently assessed by two reviewers using the Cochrane Risk of Bias tool (RoB 1.0) as described in the Cochrane Handbook. Each study was evaluated across seven domains: random sequence generation, allocation concealment, blinding of participants and personnel, blinding of outcome assessment, incomplete outcome data, selective reporting, and other sources of bias. The risk of bias for each domain was judged as low, high, or unclear. Any disagreements were resolved through discussion with a third reviewer.

### 2.6 Statistical analysis

Statistical analyses were performed using RevMan 5.4 software (Cochrane Collaboration). Continuous outcomes were pooled using mean difference (MD) with 95% confidence intervals (CI), while dichotomous outcomes were analyzed using odds ratios (OR) with 95% CI. Statistical significance was set at P < 0.05. Heterogeneity was assessed using the I^2^ statistic. A fixed-effect model was applied when heterogeneity was low (I^2^ ≤ 50%); otherwise, a random-effects model was used. Sensitivity or subgroup analyses were conducted when appropriate to explore potential sources of heterogeneity. Publication bias was assessed qualitatively using funnel plot symmetry when sufficient studies were available.

## 3 Results

### 3.1 Literature search results

**FIGURE 1:**
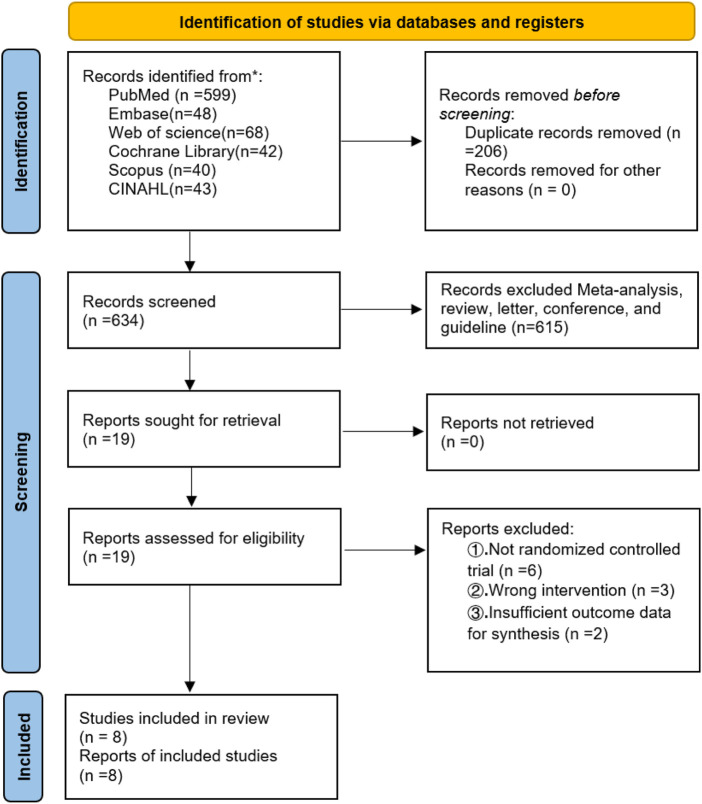
The flow diagram for the Preferred Reporting Items of Systematic Reviews and Meta-Analyses (PRISMA).

A total of 840 records were identified from electronic databases, including PubMed (n = 599), Embase (n = 48), Web of Science (n = 68), the Cochrane Library (n = 42), Scopus (n = 40), and CINAHL (n = 43). After removing 206 duplicate records, 634 records remained for title and abstract screening, of which 615 were excluded. The full texts of 19 reports were sought and successfully retrieved (0 reports not retrieved) and subsequently assessed for eligibility. Of these, 11 reports were excluded for the following reasons: not a randomized controlled trial (n = 6), wrong intervention (n = 3), and insufficient outcome data for synthesis (n = 2). Finally, 8 studies (8 reports) were included in the systematic review and meta-analysis (Figure 1). The PRISMA 2020 checklist is provided in the Supplementary Material.

### 3.2 Study characteristics and risk of bias

**FIGURE 2:**
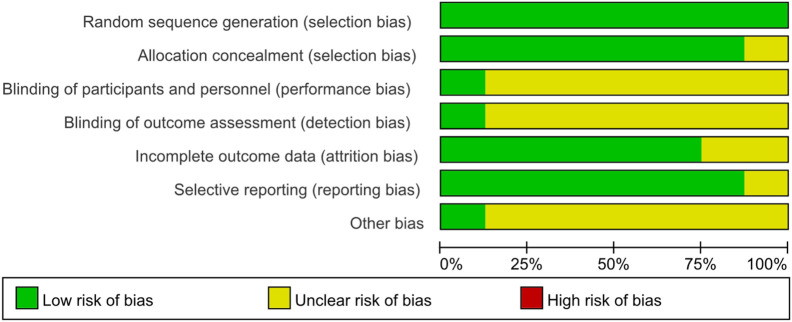
Bias risk maps for every project that is featured.

**FIGURE 3:**
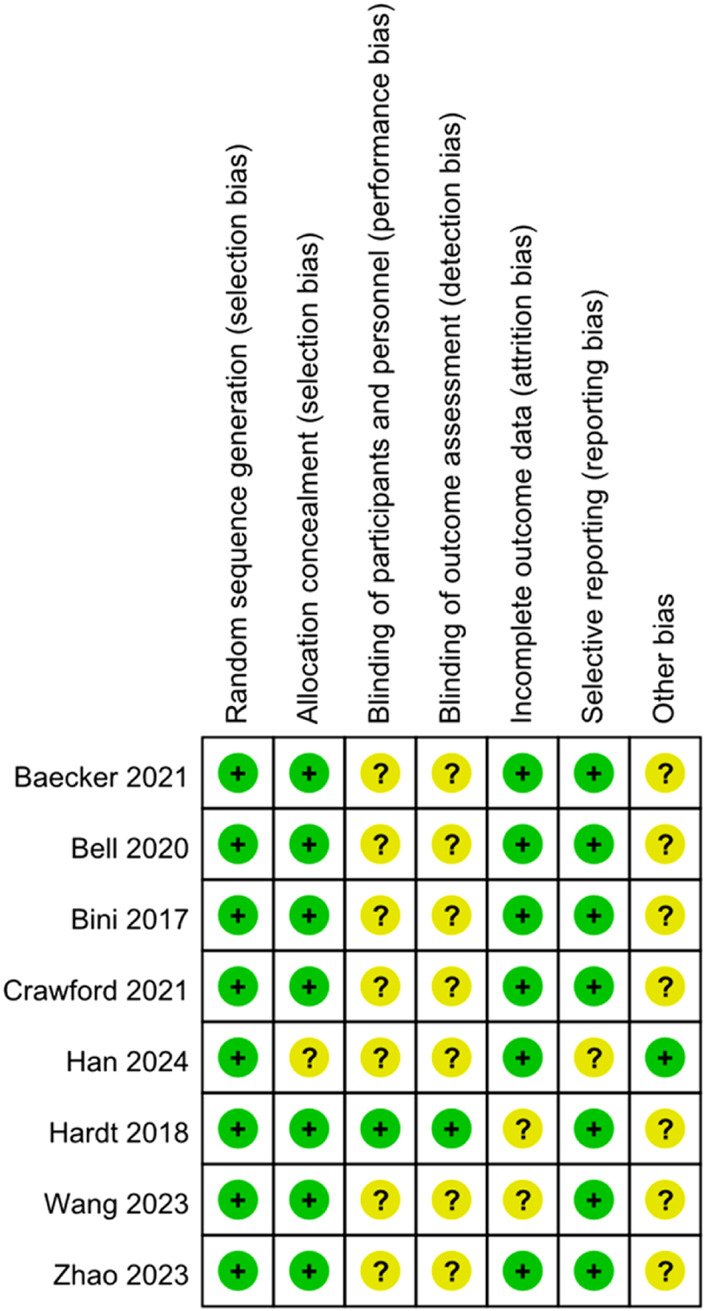
Map of bias risk.

Risk of bias was assessed for all included studies using the Cochrane Risk of Bias tool (RoB 1.0). All eight included studies were randomized controlled trials, as summarized in Table 2. Overall, the methodological quality of the included trials was judged to be moderate. Most studies reported adequate random sequence generation; however, details regarding allocation concealment were frequently insufficient or unclear, resulting in an unclear risk of selection bias in several trials. Due to the nature of rehabilitation interventions, blinding of participants and personnel was generally not feasible and was therefore judged as unclear risk in most studies. Blinding of outcome assessment was variably reported, leading to mixed judgments across studies. Incomplete outcome data and selective reporting were generally judged as low risk, as most trials reported acceptable follow-up rates and prespecified outcomes. Other sources of bias were considered low or unclear in the majority of studies. A detailed summary of the risk of bias assessment for each domain and each study is presented in Figures 2, 3. Risk of bias was assessed at the study level rather than on an outcome-specific basis, as the RoB 1.0 tool is designed to evaluate trial-level methodological domains that typically apply across outcomes within the same randomized study.

**TABLE 2:** Fundamental details of the literature contained.

Author (year)	Country	Sample (size)		Age (years)		Study type
		APP group	Traditional group	APP group	Traditional group	
Bäcker (2021)	Germany	20	15	62.95 ± 8.25	66.27 ± 10.57	RCT
Bell (2020)	USA	12	12	64.00 ± 7.70	65.30 ± 8.30	RCT
Bini (2017)	USA	14	15	62.9	63.6	RCT
Han (2024)	Korea	14	14	66.38 ± 7.26	69.54 ± 6.63	RCT
Crawford (2021)	USA	160	185	63.20 ± 8.60	64.50 ± 8.90	RCT
Hardt (2018)	Germany	22	*25*	63.3 ± 8.00	67.6 ± 10.20	RCT
Wang (2023)	Chinese	43	43	68	70	RCT
Zhao (2023)	Chinese	50	50	65	65	RCT

### 3.3 Meta analysis results

#### 3.3.1 VAS

**FIGURE 4:**
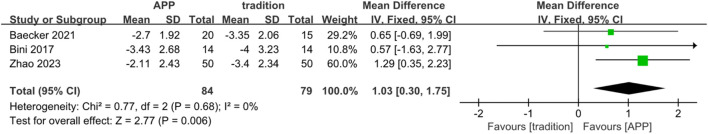
Forest maps of APP and standard 3-month VAS scores.

Three studies reported pain using the visual analogue scale (VAS) at 3 months after surgery. Lower VAS scores indicate less pain. No heterogeneity was observed (Chi^2^ = 0.77, df = 2, P = 0.68; I^2^ = 0%); therefore, a fixed-effect model was applied. The pooled analysis showed a statistically significant between-group difference (MD = 1.03, 95% CI 0.30 to 1.75; P = 0.006), indicating lower pain scores in the app-based rehabilitation group compared with the traditional rehabilitation group (Figure 4).

#### 3.3.2 10-M walk test

**FIGURE 5:**
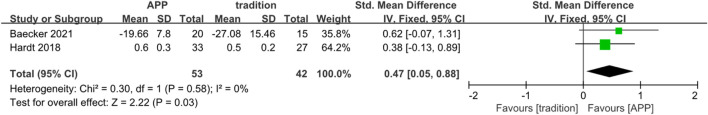
Forest map comparing APP and traditional groups’ 10-m walking test 1 week after surgery.

Two studies reported early postoperative 10-m walk performance. Because the outcome was reported as walking time (s) in one trial and walking speed (m/s) in another, results were synthesized using the standardized mean difference (SMD), with direction aligned so that higher values indicated better walking performance. No heterogeneity was observed (I^2^ = 0%, P = 0.58); therefore, a fixed-effect model was applied. The pooled analysis showed a statistically significant improvement in the app-based rehabilitation group compared with traditional rehabilitation (SMD = 0.47, 95% CI 0.05 to 0.88; P = 0.03) (Figure 5).

#### 3.3.3 Timed Up and Go score

**FIGURE 6:**
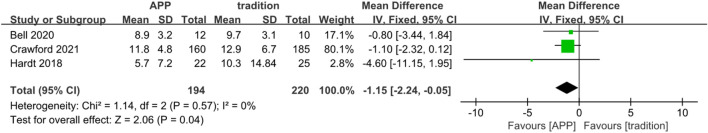
Forest map comparing APP and traditional groups’ “stand up and walk” timing test more than 1 week after surgery.

Four studies reported physical performance using the Timed Up and Go (TUG) test at ≥1 week postoperatively. Heterogeneity was low (I^2^ = 19%, P = 0.30), and a fixed-effect model was used. The pooled results indicated a significant difference between groups (MD = −1.75, 95% CI −2.55 to −0.94; P < 0.0001) (Figure 6).

#### 3.3.4 ROM

**FIGURE 7:**
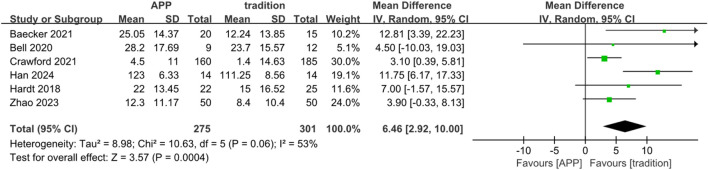
Forest map comparing knee joint range of motion scores of APP group and traditional group more than 1 week after surgery.

Six studies reported knee range of motion (ROM) at more than 1 week after surgery. Moderate heterogeneity was observed (Chi^2^ = 10.63, df = 5, P = 0.06; I^2^ = 53%); therefore, a random-effects model was applied. The pooled analysis showed that the app-based rehabilitation group achieved a significantly greater ROM than the traditional rehabilitation group (MD = 6.46°, 95% CI 2.92 to 10.00; P = 0.0004) (Figure 7).

#### 3.3.5 KSS

**FIGURE 8:**
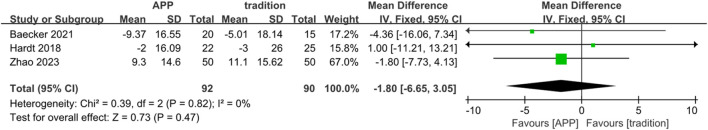
Forest map of knee association score (KSS) compares APP and traditional groups longer than 1 week after surgery.

Three studies reported the Knee Society Score (KSS) at ≥2 weeks after surgery. No heterogeneity was detected (I^2^ = 0%, P = 0.82), and a fixed-effect model was used. No statistically significant difference was observed between groups (MD = −1.48, 95% CI −6.65 to 3.05; P = 0.47) (Figure 8).

## 4 Discussion

Based on the pooled results, the effects of app-based digital rehabilitation after TKA varied across different outcome domains. Pain was assessed using the Visual Analogue Scale (VAS), a patient-reported outcome in which lower scores indicate less pain (Huskisson, 1974). At 3 months postoperatively, the app-based rehabilitation group demonstrated significantly better pain relief, suggesting that digital programs may support sustained symptom control during mid-term recovery. Knee function was evaluated using both subjective and objective measures. The Knee Society Score (KSS), which reflects overall knee status and functional ability, showed no significant difference between groups, indicating that app-based rehabilitation did not lead to measurable advantages in composite knee scores at the assessed follow-up (Insall et al., 1989). In contrast, performance-based functional outcomes favored the app group. The Timed Up and Go (TUG) test, which reflects dynamic balance and basic mobility, and the 10-m Walk Test, which evaluates short-distance walking ability, both showed superior results with digital rehabilitation, suggesting earlier improvement in mobility-related tasks (Podsiadlo and Richardson, 1991; Peters et al., 2013).

Knee range of motion (ROM), measured in degrees and reflecting postoperative joint flexibility, was also greater in the app-based group beyond 1 week, indicating a potential benefit of guided, repeated exercises on early joint mobility. Although the improvement in ROM was statistically significant, its clinical relevance should be interpreted cautiously. The pooled mean difference of approximately 6° likely reflects early postoperative gains in joint mobility, which may facilitate early functional activities (Stratford et al., 2010; Mutsuzaki et al., 2017). However, it remains uncertain whether this magnitude of improvement translates into sustained long-term functional benefits (Köglberger et al., 2022).

The lack of a significant difference in the Knee Society Score (KSS) may be partly explained by the composite nature and inherent limitations of this outcome measure, which has been reported to be less sensitive to nuanced or domain-specific postoperative changes (Leica et al., 2024). In addition, clinical scores like the KSS rely on comprehensive clinician-assessed domains, including physical examination and functional tasks, which may be less influenced by remotely delivered interventions and less capable of capturing specific improvements facilitated by app-based rehabilitation. Overall, these findings suggest that while app-based rehabilitation may not substantially alter composite knee function scores, it may offer potential benefits in pain relief, mobility performance, and early recovery of knee motion during the early to mid-term postoperative period.

In addition to evidence from post-TKA rehabilitation, mobile application–based and other digital interventions have been evaluated in broader knee conditions. For instance, in knee osteoarthritis populations, interactive mobile applications have been shown to improve exercise accuracy and several clinical outcomes, including daily activity and quality of life, compared with handout-based programs. (Thiengwittayaporn et al., 2023). Systematic reviews also suggest that mobile apps incorporating behavior change techniques may enhance exercise adherence in hip and knee osteoarthritis, which is critical for achieving long-term functional gains (Hinman et al., 2022). Moreover, digitally delivered exercise interventions for knee osteoarthritis have been found to be comparable to face-to-face programs for pain, function, and quality of life, indicating that digitally supported exercise may be a viable alternative to conventional delivery in chronic knee disorders (Fan et al., 2024).

Beyond degenerative conditions, digital applications have also been studied in other knee rehabilitation contexts. A recent systematic review of digital applications in anterior cruciate ligament reconstruction suggested that digital tools can achieve similar or superior outcomes in patient-reported measures, muscle strength, and range of motion compared with standard physiotherapy, and may enhance engagement and adherence to rehabilitation plans (Fricke et al., 2025). Similarly, mobile health interventions following ACL reconstruction have shown benefits in knee function and symptoms (Guo et al., 2023). These findings support the broader applicability of mobile app–based or digital rehabilitation approaches across multiple knee conditions, and help contextualize our findings in TKA within a wider evidence base.

These domain-specific effects provide a basis for discussing the advantages and limitations of mobile app–based rehabilitation in patients after TKA, in comparison with other rehabilitation approaches. Compared with traditional face-to-face rehabilitation, mobile app–based interventions offer greater flexibility and accessibility by enabling patients to perform guided exercises at home, which may be particularly beneficial for those with limited access to outpatient services. (Wang et al., 2023; Buhagiar et al., 2019). In contrast to other telerehabilitation approaches that rely heavily on real-time supervision, app-based programs can deliver standardized exercise content and reminders in a scalable manner, potentially supporting adherence and continuity of rehabilitation (Wang et al., 2023; Pritwani et al., 2024). These features may help explain the observed advantages in pain relief and performance-based mobility outcomes in the present analysis. However, limitations should also be acknowledged. The lack of a significant difference in composite knee scores suggests that app-based rehabilitation may not fully substitute for supervised rehabilitation when individualized manual guidance and immediate correction are required (Tsang et al., 2024; Buhagiar et al., 2019). In addition, variability in application design, exercise intensity, and user engagement may contribute to heterogeneous effects across studies (Pritwani et al., 2024). Compared with hybrid rehabilitation models that incorporate wearable devices or in-person supervision, standalone mobile applications may provide limited objective monitoring, which could influence long-term functional outcomes (King et al., 2023). Therefore, mobile app–based rehabilitation appears to be a valuable adjunct or alternative to existing rehabilitation strategies after TKA, rather than a universal replacement for other rehabilitation modalities.

Moreover, access to postoperative rehabilitation after TKA may be limited for many patients due to geographical distance and early discharge, particularly for those who are not local residents (Wainwright et al., 2021; French et al., 2025). In this context, digital rehabilitation provides a feasible means of delivering structured rehabilitation guidance at home and may help maintain continuity of care after hospital discharge. Although TKA is an effective treatment for end-stage knee osteoarthritis, adequate postoperative rehabilitation remains essential for functional recovery (Carr et al., 2012). Consistent with this rationale, the present meta-analysis showed that app-based digital rehabilitation was associated with better pain relief and improved mobility-related outcomes compared with conventional rehabilitation. Early postoperative rehabilitation is commonly regarded as an important period for restoring knee range of motion and functional capacity, which may facilitate subsequent individualized strengthening and functional training (Shakespeare and Kinzel, 2005). Importantly, our findings add to the existing evidence by providing a focused synthesis of mobile app–based rehabilitation after TKA and showing that its benefits may be outcome-specific, with clearer improvements in pain, mobility performance and ROM than in broader composite patient-reported scores. Clinically, these results support considering app-based rehabilitation as an adjunct or alternative for patients with limited access to in-person therapy, while emphasizing the need for standardized outcome reporting and longer follow-up in future trials.

### 4.1 Limitations of the study

This meta-analysis has several limitations that should be considered when interpreting the findings. First, although a predefined subgroup analysis based on different control interventions was planned, the limited number of eligible studies and insufficient data prevented meaningful subgroup analyses, which may restrict the depth of interpretation and render some conclusions preliminary. This constraint may have reduced the depth of interpretation and rendered some findings preliminary. In addition, sensitivity analyses were performed where appropriate to explore potential sources of heterogeneity; however, residual heterogeneity could not be fully explained, likely reflecting clinical and methodological variability across studies.

Second, there was a lack of standardized postoperative rehabilitation protocols after total knee arthroplasty, with considerable variation in intervention content, intensity, duration, and follow-up time among the included trials. Such heterogeneity may have influenced the pooled effect estimates, particularly for outcomes such as knee range of motion.

Third, several included trials had relatively small sample sizes, which may have limited statistical power and increased the risk of imprecise effect estimates. Furthermore, blinding of participants and personnel was generally not feasible due to the nature of rehabilitation interventions. This inherent limitation may have introduced performance and detection bias and should be considered when interpreting the results.

Finally, while only randomized controlled trials were included to enhance internal validity and reduce bias, this strict inclusion criterion may limit the generalizability of the findings. Observational studies and real-world evidence reflecting routine clinical practice were not considered, and such studies could provide additional insights. Additionally, research on app-based digital rehabilitation after TKA remains limited in certain regions. Further well-designed randomized controlled trials, as well as pragmatic studies with standardized intervention protocols, consistent outcome measures, and longer follow-up periods, are warranted to strengthen the evidence base.

### 4.2 Implications for practice and certainty of evidence (GRADE)

The Grading of Recommendations Assessment, Development and Evaluation (GRADE) approach, the certainty of evidence for the outcomes assessed in this meta-analysis was judged to range from low to moderate (Guyatt et al., 2008). As all included studies were randomized controlled trials, the initial certainty of evidence was considered high; however, downgrading was applied due to methodological limitations and variability among studies.

Specifically, the certainty of evidence for pain reduction, assessed using the Visual Analogue Scale (VAS), and for performance-based functional outcomes, including the Timed Up and Go test and the 10-m Walk Test, was rated as moderate. This rating was supported by consistent effect estimates, low statistical heterogeneity, and clinically meaningful improvements favoring app-based digital rehabilitation. In contrast, the certainty of evidence for knee range of motion (ROM) and composite knee function scores (Knee Society Score, KSS) was rated as low to moderate, primarily due to unclear allocation concealment, lack of blinding inherent to rehabilitation interventions, and heterogeneity in intervention protocols and follow-up durations across studies.

Based on the available evidence, app-based digital rehabilitation can be conditionally recommended as an adjunct or alternative to conventional rehabilitation following total knee arthroplasty, particularly for improving postoperative pain and early functional mobility in patients with limited access to face-to-face physiotherapy. However, the overall strength of recommendation remains conditional, and further high-quality randomized controlled trials with standardized intervention protocols, consistent outcome measures, and longer follow-up periods are required to increase the certainty of evidence and support stronger clinical recommendations.

## 5 Conclusion

In conclusion, this meta-analysis suggests that mobile app–based digital rehabilitation after total knee arthroplasty may provide advantages over traditional rehabilitation in specific outcome domains. App-based rehabilitation was associated with improved pain relief, superior performance-based functional outcomes, and greater early postoperative gains in knee range of motion. However, no significant improvement was observed in composite knee function scores such as the Knee Society Score.

Importantly, the certainty of evidence ranged from low to moderate, reflecting methodological limitations, variability in intervention protocols, and differences in follow-up durations among the included trials. Therefore, mobile app–based rehabilitation should be regarded as a potentially useful adjunct or alternative to conventional rehabilitation, particularly for patients with limited access to in-person physiotherapy. Further well-designed randomized controlled trials with standardized outcome measures and longer follow-up periods are required to strengthen the evidence base and clarify the long-term clinical effectiveness of app-based digital rehabilitation after total knee arthroplasty.

## Data Availability

The raw data supporting the conclusions of this article will be made available by the authors, without undue reservation.
